# Crystallisation in Melts of Short, Semi-Flexible Hard-Sphere Polymer Chains: The Role of the Non-Bonded Interaction Range

**DOI:** 10.3390/e21090856

**Published:** 2019-09-01

**Authors:** Timur Shakirov

**Affiliations:** Institute of Physics, Martin-Luther-University, 06099 Halle, Germany; timur.shakirov@physik.uni-halle.de

**Keywords:** hard sphere, polymer, semi-flexible polymer, polymer melt, phase transition, entropy, rotator phase

## Abstract

A melt of short semi-flexible polymers with hard-sphere-type non-bonded interaction undergoes a first-order crystallisation transition at lower density than a melt of hard-sphere monomers or a flexible hard-sphere chain. In contrast to the flexible hard-sphere chains, the semi-flexible ones have an intrinsic stiffness energy scale, which determines the natural temperature scale of the system. In this paper, we investigate the effect of weak additional non-bonded interaction on the phase transition temperature. We study the system using the stochastic approximation Monte Carlo (SAMC) method to estimate the micro-canonical entropy of the system. Since the density of states in the purely hard-sphere non-bonded interaction case already covers 5600 orders of magnitude, we consider the effect of weak interactions as a perturbation. In this case, the system undergoes the same ordering transition with a temperature shift non-uniformly depending on the additional interaction. Short-range attractions impede ordering of the melt of semi-flexible polymers and decrease the transition temperature, whereas relatively long-range attractions assist ordering and shift the transition temperature to higher values, whereas weak repulsive interactions demonstrate an opposite effect on the transition temperature.

## 1. Introduction

Hard-sphere polymer models are easy in formulation and thus widely used for the investigation of the thermodynamic properties of polymeric systems. The simplest model is a system of flexible chains of tangent hard-sphere beads. Fixed bond length reduces the entropy of the system and shifts the crystallisation volume fraction to a higher value, $\phi_{c}\approx 0.51$ [1,2], than the corresponding volume fraction of non-bonded hard spheres $\phi_{c}=0.494$ [3]. However, the entropy reduction does not affect the structure of the ordered state, both systems form a monomer-based mixture of face-centred cubic (FCC) and hexagonal close packed (HCP) regions [1,2,3]. The role of square-well attraction was investigated for single flexible hard-sphere chains theoretically (for short chains) [4] and numerically [5,6]. At low temperatures, long chains form a stable crystal structure in the core of the frozen globule. The type of the crystal structure, FCC, HCP, or body-centred cubic (BCC), depends on the chain length and interaction width of the square-well potential [6]. Unlike the models discussed above, typical polymers are semi-flexible, and the local stiffness plays an important role in the folding and crystallisation of polymers [7,8,9,10,11,12,13]. For instance, a geometrical stiffness, where the bead size is bigger than the bond length, induces the formation of complex non-crystalline single-chain structures depending on the bead size to the bond length ratio [14].

The semi-flexible tangent hard-sphere chain is a simplified model of real polymers. We calculated previously, by means of stochastic approximation Monte Carlo (SAMC) simulation [15,16,17], the micro-canonical entropy of a dense semi-flexible tangent hard-sphere polymer system [18] (we provide a more detailed description of the model in Section 2). When the system is big enough, the model polymer melt undergoes an ordering transition into a layered rotator-like phase, which is similar to the rotator phases of n-alkanes: a family of smectically ordered phases with different ordering in layers [19,20,21,22]. However, for a small hard-sphere chain system the size effects disorder the layered structure, and the system forms a nematic phase with a hexatic ordering in the plain perpendicular to the director [18].

We focus in the next sections on the influence of weak non-bonded interactions on the thermodynamic properties of the semi-flexible hard-sphere polymer system. The micro-canonical entropy of the system reaches the maximal difference $\Delta S/k_{B}\approx$ 12,960 ($k_{B}=1$ is the Boltzmann factor). Thus, the density of states covers ($g_{\max}/g_{\min}=e^{\Delta S}$) more than 5600 orders of magnitude [18]. The estimation of the micro-canonical entropy requires an extremely long computational time for such a system. Therefore, we focus on corrections to the entropy caused by weak square-well attraction or square-shoulder repulsion, which can be observed during a productive run with the micro-canonical entropy of the system having purely hard-sphere non-bonded interactions. The entropy estimation remains unchangeable during the productive run, which makes it possible to accumulate correct arithmetic averages and distributions of parameters of the system.

## 2. Materials and Methods

We consider a system of hard-sphere polymer chains having N=10 tangent beads of unit diameter d=1. The system is composed of $N_{c}=720$ polymers in a simulation box with periodic boundary conditions. The volume fraction is $\phi =\left(\pi /6\right)d^{3}\cdot NN_{c}/L^{3}\approx 0.47$, with L=20d being the linear size of the simulation box, which is below the crystallisation value $\phi_{c}=0.494$ for a system of non-bonded hard-spheres [3]. The leading interactions are hard-sphere-type interactions of non-bonded beads:

(1) $$U_{\mathrm{nb}}\left(r\right)=\left\{\begin{array}{cc} \infty & r<d\\ 0 & r\geq d \end{array}\right.,$$

where *r* is the separation between the centres of two beads, and a square-well bond-angle potential

(2) $$U_{a}\left(\theta \right)=\left\{\begin{array}{cc} -\varepsilon & \theta \leq \theta_{s}\\ 0 & \theta >\theta_{s} \end{array}\right.,$$

where θ is the angle formed by adjacent bonds along a chain and $\cos\theta_{s}=0.9$ or $\theta_{s}\approx 26\;\deg$. The non-bonded interaction (1) restricts accessible configurational space, but has no energy contribution. In contrast, the bond-angle interaction (2) defines the energy of the system $U=\sum U_{a}=-n_{a}\varepsilon$, with $n_{a}$ being the number of bond-angles in the range $\theta \leq \theta_{s}$.

We numerically estimate the entropy, $S\left(U\right)$, of the system by the SAMC method [15,16,17,18]. The micro-canonical entropy defines the logarithm of the density of states (DOS) of the system $\ln g\left(U\right)=S\left(U\right)$. The SAMC method is a variant of the Wang–Landau algorithm having, in contrast to the original procedure, proven convergence to the exact DOS [16,17]. When the micro-canonical entropy is known, we can calculate any thermodynamic property of the system after accumulation of corresponding averages during a productive run with the fixed DOS [18]. The value of a thermodynamical parameter *A* at canonical temperature *T* can then be calculated as

(3) $$\langle A\rangle \left(T\right)=\frac{1}{Z\left(T\right)}\sum_{n_{a}}\bar{A}\left(n_{a}\right)e^{S\left(n_{a}\right)-U/T},$$

where $Z\left(T\right)=\sum_{n_{a}}e^{S-U/T}$ is partition function and $\bar{A}\left(n_{a}\right)$ is the mean value of *A* at energy $U=-n_{a}\varepsilon$, which can be estimated as an arithmetical average accumulated during a productive run. As an example, we calculated the canonical heat capacity $C_{V}$:

(4) $$C_{V}\left(T\right)=\frac{\langle U^{2}\rangle \left(T\right)-{\langle U\rangle }^{2}\left(T\right)}{T^{2}}$$

**Figure 1:**
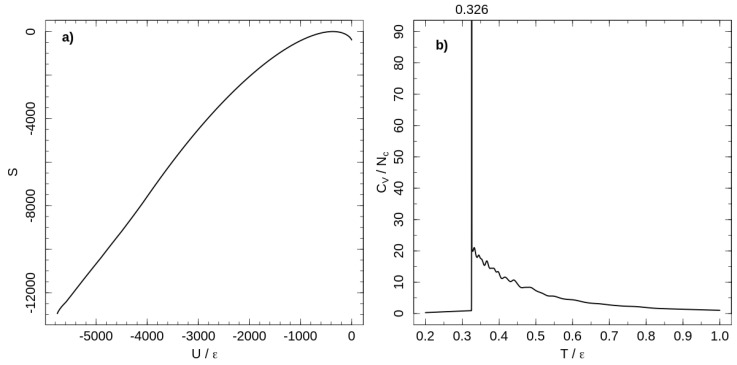
(**a**) micro-canonical entropy for the system of tangent hard-sphere flexible polymers with purely repulsive non-bonded interaction. Entropy shifted according to its maximal value. (**b**) Temperature dependence of the heat capacity per chain calculated with the entropy shown in the left panel.

The temperature dependence of the heat capacity is shown in Figure 1. A first-order phase transition at T=0.326ε separates ordered and liquid-like disordered states of the system. Chains are flexible at high temperatures, but decreasing the temperature increases chain stiffness and induces the first-order phase transition from a disordered melt to a rotator-like phase [18]. In the rotator-like phase, all chains are stretched along a common director and hexatically ordered in a cross section perpendicular to the director. This phase transition is a lyotropic one and occurs as the Kuhn length reaches a critical value, depending on polymer concentration [18].

Non-bonded interactions, square-well attraction and square-shoulder repulsion, can be defined in a general way:

(5) $$U_{\mathrm{nb}}\left(r\right)=\left\{\begin{array}{cc} \infty & r<d\\ -\varepsilon_{c} & d\leq r<d+\delta \\ 0 & d+\delta \leq r \end{array}\right.,$$

with positive $\varepsilon_{c}>0$ corresponding to the attractive square-well potential, and $\varepsilon_{c}<0$ to the repulsive square-shoulder interaction. The total energy of the system now depends on both the bond-angle and non-bonded potentials:

(6) $$U=-n_{a}\cdot \varepsilon -n_{c}\cdot \varepsilon_{c},$$

with $n_{c}$ being the number of bead pair separations within the non-bonded interaction range. We take the pair of parameters $\left(n_{a},n_{c}\right)$ as the parameters describing a macro-state of the system. For the set of these macro-states, we calculate a two-dimensional (2D) entropy $S\left(n_{a},n_{c}\right)$ and corresponding 2D DOS $g\left(n_{a},n_{c}\right)=e^{S\left(n_{a},n_{c}\right)}$. The 2D DOS $g\left(n_{a},n_{c}\right)$ is related to the 1D DOS $g\left(U\right)\equiv g\left(n_{a}\right)=\sum_{n_{c}}g\left(n_{a},n_{c}\right)$. On the other hand, for each macro-state $\left(n_{a},n_{c}\right)$, a conditional probability $p\left(n_{c}|n_{a}\right)=g\left(n_{a},n_{c}\right)/g\left(n_{a}\right)$ can be calculated and so

(7) $$g\left(n_{a},n_{c}\right)=p\left(n_{c}|n_{a}\right)\cdot g\left(n_{a}\right).$$

The 2D DOS as well as the conditional probabilities are a priori unknown and should be calculated during a simulation. However, since even the estimation of a 1D DOS $g\left(n_{a}\right)$ requires a long computational time, an estimation of a 2D DOS similar to [23,24] is practically impossible for this model. We perform a productive run with the fixed 1D entropy $S\left(n_{a}\right)$ instead of the time-expensive SAMC simulation and accumulate a visitation histogram $H\left(n_{a},n_{c}\right)$. We base our estimation of the conditional probabilities on the accumulated histograms:

(8) $$p\left(n_{c}|n_{a}\right)\approx \frac{H\left(n_{a},n_{c}\right)}{\sum_{n_{c}|n_{a}}H\left(n_{a},n_{c}\right)},$$

where $\sum_{n_{c}|n_{a}}$ indicates a sum over all number of contacts observed for a given $n_{a}$. The disadvantage of this method is the concentration of reachable $n_{c}$ values around the most probable value corresponding to a given $n_{a}$ value. Owing to that we can observe only $n_{c}$ values having a probability of a few orders of magnitude less than the maximal one for a given $n_{a}$. In essence, we assume that configurations are very little affected by a weak non-bonded interaction $|\varepsilon_{c}|\ll \varepsilon$ and consider it as a perturbation. This assumption fails close to the minimal bond-angle energy, since the total configurational energy variation is mainly caused in this case by contact changes. Thus, we are limited in our analysis to the states located far enough from the ground state in the phase space.

The two-dimensional histogram $H\left(n_{a},n_{c}\right)$ has a clear geometrical meaning: number of bead pairs separated by a distance in the range d≤r<d+δ observed during a productive run. This number depends neither on the energy associated with each contact nor, in particular, the sign of the energy contribution. Consequently, the square-well attraction and square-shoulder repulsion interactions of the same width produce the same visitation histogram. This allows us to use the accumulated histogram for the both types of models. As we mentioned above, in the following, we associate attractive interactions with positive values of $\varepsilon_{c}$ and repulsive with negative ones.

## 3. Results

### 3.1. Structural Properties of the Non-Perturbed System

**Figure 2:**
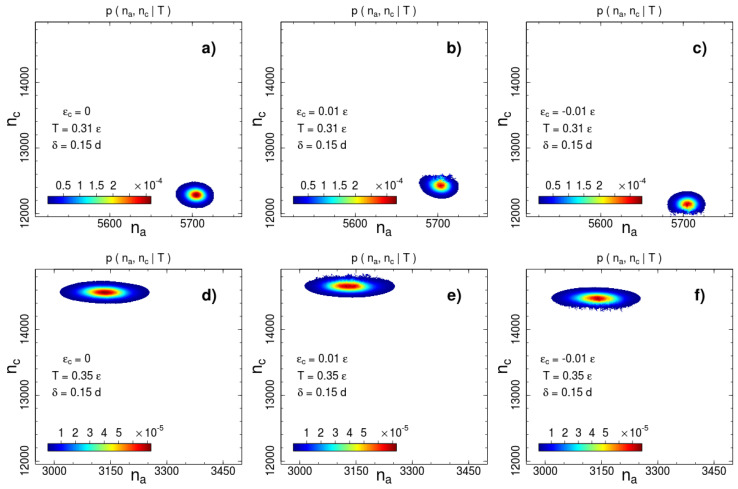
Canonical probability distribution for the potential width δ=0.15d at two temperatures: (**a**–**c**) T=0.31ε and (**d**–**f**) T=0.35ε. Columns from left to right correspond to the cases of no additional interactions, square-well attraction $\varepsilon_{c}=0.01\varepsilon$ and square-shoulder repulsion $\varepsilon_{c}=-0.01\varepsilon$. Probabilities less than than 1% of the maximal value are not shown.

**Figure 3:**
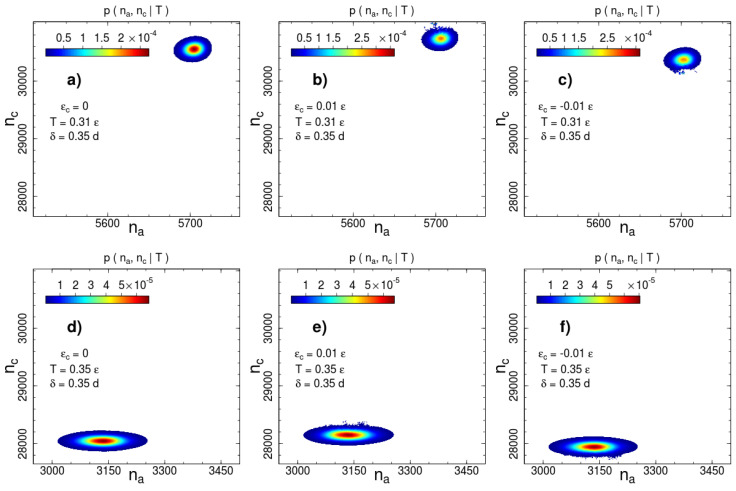
Canonical probability distribution for the potential width δ=0.35d at two temperatures: (**a**–**c**) T=0.31ε and (**d**–**f**) T=0.35ε. Columns from left to right correspond to the cases of no additional interactions, square-well attraction $\varepsilon_{c}=0.01\varepsilon$ and square-shoulder repulsion $\varepsilon_{c}=-0.01\varepsilon$. Probabilities less than than 1% of the maximal value are not shown.

On the basis of the conditional probabilities $p\left(n_{c}|n_{a}\right)$ estimated according to (8), we calculate the 2D entropy $S\left(n_{a},n_{c}\right)=S\left(n_{a}\right)+\ln p\left(n_{c}|n_{a}\right)$. We provided calculations for a series of interaction widths from δ=0.025d to δ=0.35d with a step 0.025d as well as for δ=0.01d and δ=0.5d. Some examples of canonical probabilities of macro-states $\left(n_{a},n_{c}\right)$,

(9) $$p\left(n_{a},n_{c}|T\right)=\frac{1}{Z\left(T\right)}e^{S\left(n_{a},n_{c}\right)-U\left(n_{a},n_{c}\right)/T},$$

are shown in Figure 2 and Figure 3 for two interaction ranges δ=0.15 and δ=0.35 and two temperatures: below (T=0.31ε) and above (T=0.35ε) the phase transition temperature $T_{c}=0.326\varepsilon$. The most probable number of contacts significantly changes during the phase transition for both interaction ranges, but the direction of the change is opposite. The number of short-range interacting pairs decreases during ordering (Figure 2), whereas for the long-range case we observe an increase in the number of contacts (Figure 3). This difference indicates a rearrangement of the local packing of beads. We analyse the local rearrangement by means of the radial distribution function

(10) $$g_{2}\left(r\right)=\frac{\rho \left(r\right)}{\rho },$$

where $\rho \left(r\right)$ and ρ are monomer density at a distance *r* from a given bead averaged over all beads and the mean monomer density in the simulation box, respectively.

The radial distribution function can be estimated without further simulations with the help of the mean number of contacts $\langle n_{c}\left(\delta \right)\rangle \left(T\right)$ observed at temperature *T* for the interaction range δ. We calculate the mean values based on the 2D entropy function similar to (3) by replacing the summation index by the 2D macro-state identifier $\left(n_{a},n_{c}\right)$ and using the total energy (6). We take the contact energy parameter $\varepsilon_{c}=0$ to analyse a non-perturbed radial distribution function describing the same system as in [18]. Since the difference of $\langle n_{c}\left(\delta_{k}\right)\rangle -\langle n_{c}\left(\delta_{k-1}\right)\rangle$ is the mean number of beads pairs located in the spherical layer $\delta_{k-1}<r<\delta_{k}$, the local density of monomers in the layer confined by two next interaction ranges $\delta_{k-1}$ and $\delta_{k}$ can be estimated as

(11) $$\rho \left(r_{k}\right)=\rho \left(\frac{\delta_{k-1}+\delta_{k}}{2}\right)\approx \frac{3}{2\pi NN_{c}}\frac{\langle n_{c}\left(\delta_{k}\right)\rangle -\langle n_{c}\left(\delta_{k-1}\right)\rangle }{\delta_{k}^{3}-\delta_{k-1}^{3}}.$$

**Figure 4:**
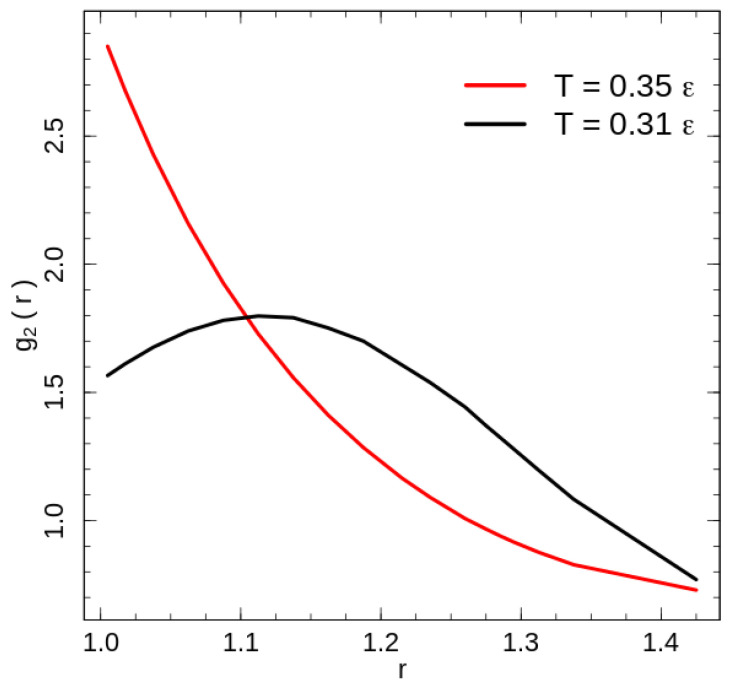
The radial distribution function calculated for two temperatures: T=0.31ε (black line) and T=0.35ε (red line).

The calculated radial distribution function for the system with purely repulsive non-bonded interaction is shown in Figure 4 for the two temperatures: below (T=0.31ε) and above (T=0.35ε) the phase transition temperature. Because of the fixed bond length, the bonded beads have a configuration independent contribution to the radial distribution function at r=d, hence we exclude it from the contact energy as well as radial distribution function calculations. The exclusion has only a weak effect of some value reduction of the $g_{2}\left(r\right)$ on the leftmost point $r_{1}=1.005d$ in Figure 4.

The disordered polymer liquid at temperatures above the phase transition demonstrates a radial distribution function typical for a disordered state and reaches the first minimum seemingly shortly after the rightmost point presented in Figure 4. Rotator-like ordering of stretched chains at low temperatures [18] modifies the radial distribution function. The change is caused by the combination of the global nematic ordering and the hexatic ordering in the plain perpendicular to the director. A cross section transverse to the director crosses half of the chains, i.e., $N_{c}/2=360$ chains. The packing of the 360 chains in a hexatic structure leads to a packing of 20×18 chains in a cross section with corresponding mean side lengths *d* and 10/9·d≈1.11d. Both length scales contribute the maximum of the radial distribution function below the phase transition temperature. For two beads of the nearest chains, which are shifted along the director on half of the bond length d/2, the distances for the short and long hexagon sides are $\sqrt{d^{2}+{\left(d/2\right)}^{2}}\approx 1.1d$ and $\sqrt{\left(1.1\right)d^{2}+{\left(d/2\right)}^{2}}\approx 1.2d$, respectively. Unlike the flexible chains [1,2,3,5,6], an investigation of the local structures using the common neighbour analysis [25] demonstrates very little increase in the number of atoms organised in FCC, HCP or BCC crystal structures; at low energies only ∼2% of atoms are identified as being in one of these crystal structures. This result is similar to the dense flexible polymer systems, which forms an ordered FCC/HCP crystal state at packing fractions higher than $\phi_{c}\approx 0.51$ [1,2].

### 3.2. The Role of Non-Bonded Interactions

As we mentioned above, the disadvantage of the visitation histogram accumulation is a relatively narrow range of numbers of contacts, which can be observed during a productive run. This limitation becomes apparent in Figure 2 and Figure 3. The border lines of the presented distributions correspond to contour line drawn on the level of $10^{-2}$ of the maximum of the canonical probability. A roughness of this contour line is visible even for a weak contact energy contribution with $|\varepsilon_{c}|=0.01\varepsilon$. This restricts accessible values of contact energy parameters to a very small range $|\varepsilon_{c}|∼10^{-2}\varepsilon$. In this range, the transition temperature measured in units of the contact energy is extremely high $T/\varepsilon_{c}∼10^{2}$. Thus, the contact energy cannot induce system (re)ordering, but affects thermodynamic properties such as the phase transition temperature.

**Figure 5:**
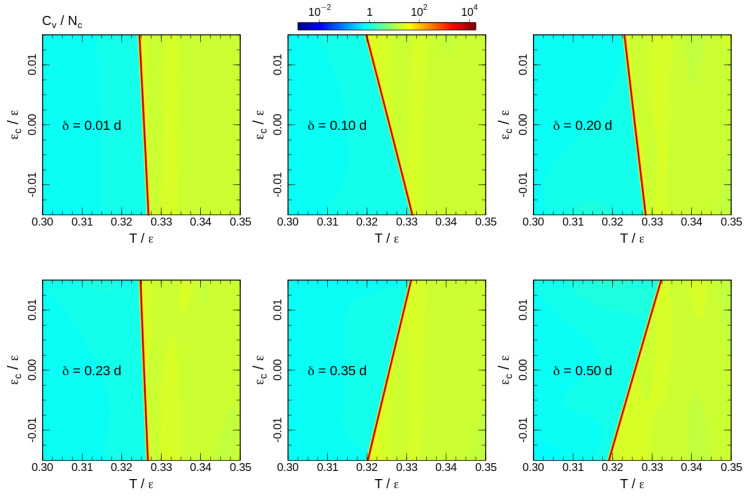
Heat capacity maps calculated for temperatures around the phase transition. The corresponding interaction ranges are shown on the maps.

**Figure 6:**
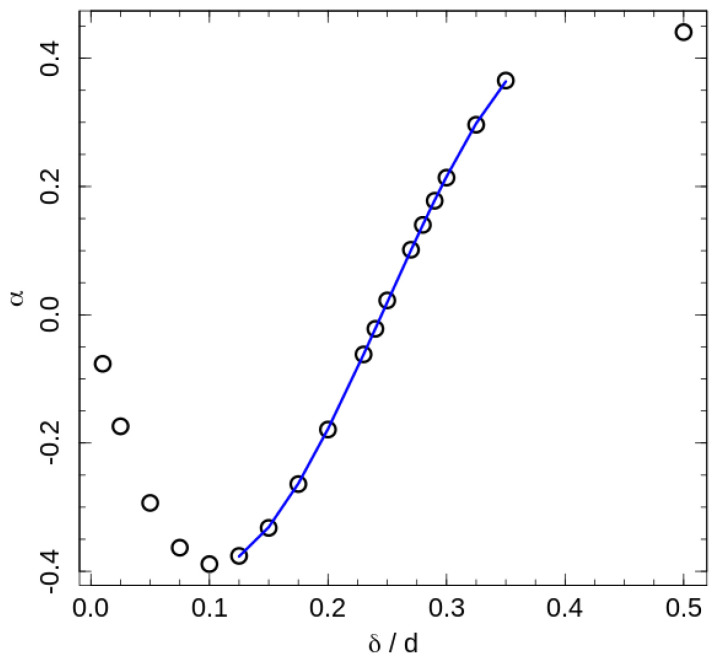
The phase transition temperature ‘shift-rate’ (12). The line represents a fit of central points by the cubic polynomial $f\left(x\right)=-0.0647-7.247x+45.662^{2}-61.310x^{3}$, with x=δ/d. The neutral interaction width estimated from the equation $f\left(x\right)=0$ is $\delta_{c}=0.245d$.

To analyse the transition temperature shift, we calculate the heat capacity for the 2D DOS similar to (4) with energy contributions of both bond-angle and non-bonded interactions (6). The heat capacity maps calculated for some interaction ranges δ are shown in Figure 5. Note that the upper halfs of the heat capacity maps ($\varepsilon_{c}>0$) correspond to the square-well attraction and the bottom halfs ($\varepsilon_{c}<0$) represent the influence of the square-shoulder repulsion. The effects of attraction and repulsion are non-uniform as a function of the interaction range. For short interaction ranges, repulsive potentials assist the ordering and increase the phase transition temperature. However, the shift of the phase transition temperature is non-uniform. The effect grows until δ=1.1d and reduces back to a level comparable to the shortest interaction range δ=1.01d for δ=1.24d. Furthermore, for longer ranges, repulsive and attractive interactions exchange roles: the repulsion impedes, whereas the attraction assists the ordering. In all cases, the effects of attractive and repulsive interactions are opposite and the phase transition temperature shift is smooth upon crossing the border-line $\varepsilon_{c}=0$. This allows a linear fit of the heat capacity maxima temperatures around the crossing point:

(12) $$T_{c}=T_{c}^{0}+\varepsilon_{c}\times \alpha \left(\delta \right),$$

where the slope $\alpha \left(\delta \right)$ reflects a ‘shift rate’ created by the additional interactions, and $T_{c}^{0}=0.326\varepsilon$ is the phase transition temperature in absence of contact energy contribution (For the fitting we considered $T_{c}^{0}$ as a free fitting parameter, but the fitted values were the same for all interaction ranges, and equal to the transition temperature of the system with purely hard-sphere interaction). Since positive values of $\varepsilon_{c}$ correspond to the square-well attraction, positive values of α>0 correspond to the increase in the transition temperature by attractive interaction, and the α<0 indicates an ordering assistance of the repulsive interaction. The fitted ‘shift-rates’ are presented in Figure 6.

The 2D hexatic ordering of the system in cross sections perpendicular to the director is governed by maximising of the 2D entropy of the chain projections onto the cross section plain [18]. This ordering is of the same nature as hexatic ordering of 2D hard disks. For perfectly aligned fully stretched chains the areal density of the projections fall into the coexistence region between disordered and hexatic phases of hard disks. We did not observe such coexistence in our data for the semi-flexible polymer model, hence we suppose an effective repulsion between neighbouring chains associated with bond-angle fluctuations. The effective repulsion induces an increase in effective areal density. An additional repulsive interaction makes for a further increase in the effective areal density, and assists ordering of the system, but only for a relative short-range interaction comparable to the typical inter-chain distances. The temperature shift-rate induced by this effect is maximal at δ≈1.1d corresponding to the longest hexagon’s side in the ordered state. Note that negative $\alpha \left(\delta \right)$ values mean that the transition is assisted by the square-shoulder repulsion, and the minimum of the $\alpha \left(\delta \right)$ indicates the strongest influence of the repulsion. Attractive interaction impedes ordering in these interaction ranges by decreasing of inter-chain distances and the effective areal density.

Upon an increase in the repulsion width, a situation is reached, where two nearest beads of neighbour chains are shifted by a distance ∼d/2 along the director. This reduces the effective areal density and the effective aspect ratio of the chains and reduces the transition temperature. In contrast, an increase in the number of close neighbours by the attractive interaction helps to increase the role of the effective repulsion and assists the ordering. Thus, the ordering assistance by attraction remains at the widest investigated interaction range. The switching of the roles of attraction and repulsion are found by the fitting of central part of the the shift-rate dependency on the interaction range by a cubic polynomial (see caption of the Figure 6). The corresponding root of the fitting polynomial is $\delta_{c}=0.245d$.

## 4. Discussion

We considered a dense semi-flexible polymer system and analysed the effects of a weak square-well attraction as well as of square-shoulder repulsion on the thermodynamic properties of the dense semi-flexible polymer system. In the case of purely hard-sphere interaction of non-bonded beads, the considered system undergoes a first-order phase transition. The driving force of this transition is the maximising of the three-dimensional orientational entropy leading to a nematic ordering of the system with synchronous hexatic ordering in the plane transverse to the nematic director. The hexatic ordering is governed by the maximising of the translational entropy contribution associated with the 2D ordering of the system in the plane transverse to the director [18].

The flexible chains crystallise in a mixture of coexisting FCC/HCP structures [1,2] as non-bonded hard spheres [3] or flexible single hard-sphere polymer chains with a square-well attraction [5,6]. While the ordered state of semi-flexible chains at the considered packing fraction demonstrates a very small total number of beads in the FCC, HCP and BCC structures. Thus, the liquid crystal ordering of the semi-flexible chains system does not necessarily lead to a crystal ordering similar to a dense system of flexible hard-sphere polymers or non-bonded hard-spheres.

Additional weak repulsive and attractive interactions have opposite effects on the phase transition temperature. When attraction impedes ordering and decreases the transition temperature, repulsion, vice versa, assists the phase transition. The square-shoulder repulsion assists the ordering transition by increasing the effective size of the chains for the interaction widths range δ<0.245d. For wider interaction ranges, the ordering transition is assisted by square-well attraction. The transition temperature shift depends not only on the interaction range, but also on the contact energy scale $\varepsilon_{c}/\varepsilon$. We analyse this dependence in terms of the first derivative on $\varepsilon_{c}$ (or slope) of the corresponding dependence of the transition temperature (12). The slope $\alpha \left(\delta \right)$ depends strongly and non-uniformly on the interaction range. The strongest transition temperature increase by repulsive interaction was observed for the interaction range comparable with the typical side length of hexagons formed in cross sections transverse to the director. After that point, the role of repulsion decreases, whereas attraction increases its influence.

A quantitative comparison of simulation results with real polymers requires a mapping of the square-well attraction on the van der Waals interaction. The analysis provided in [26] shows that δ/d∼0.4÷0.7 is necessary for mapping the interaction range. The attraction within the required range increases the transition temperatures almost equally over the whole range. The rotator-like structure of the system at low temperatures is in a good agreement with the experimental results for melts of short n-alkanes, which also undergo an ordering into one of the (smectic-like) rotator phases between melt and crystal states [19,20,21]. The temperature range of the stable rotator phases of n-alkanes is $\Delta T_{\mathrm{stable}}∼10\;K$, which corresponds to a relative temperature range of a few percents of the rotator-transition temperature $\Delta T_{\mathrm{stable}}/T_{\mathrm{rot}}∼10^{-2}$. While for the hard-sphere chain system with weak attraction, we expect crystallisation temperatures of the order $T∼\varepsilon_{c}<10^{-2}\varepsilon$, which is much less than the ordering temperature and is out of the parameter range satisfying the basic assumptions of the presented method.

## Abbreviations

The following abbreviations are used in this manuscript:

FCC	Face-centred cubic
HCP	Hexagonal close packed
BCC	Body-centred cubic
DOS	Density of states
SAMC	stochastic approximation Monte Carlo
1D	one-dimensional
2D	two-dimensional
